# Labeling tumor-associated extracellular vesicles with antibody-DNA conjugates for quantitative analysis

**DOI:** 10.3389/fmolb.2025.1531108

**Published:** 2025-01-22

**Authors:** Xiao Du, Hongxiu Li, Shiyi Shen, Chao Tian, Xiaohuan Cao, Xingang Xu, Nan Xu, Shuling Wang, Qingchang Tian

**Affiliations:** ^1^ School of Pharmacy, Hangzhou Normal University, Hangzhou, Zhejiang, China; ^2^ Key Laboratory of Elemene Class Anti-Cancer Chinese Medicines, Collaborative Innovation Center of Traditional Chinese Medicines of Zhejiang Province, Hangzhou Normal University, Hangzhou, Zhejiang, China; ^3^ Engineering Laboratory of Development and Application of Traditional Chinese Medicines, Collaborative Innovation Center of Traditional Chinese Medicines of Zhejiang Province, Hangzhou Normal University, Hangzhou, Zhejiang, China; ^4^ Laboratory of Chinese Medicine Preparation, Shandong Research Academy of Traditional Chinese Medicine, Jinan, China

**Keywords:** extracellular vesicles, antibody-DNA conjugates, Poisson distribution, proximity ligation technology, tumor diagnosis

## Abstract

**Introduction:**

Extracellular vesicles (EVs) shed from tumor cells into peripheral circulation or other body fluids are promising biomarkers for cancer diagnosis with enormously long circulation. Consequently, precise methods for differentiating normal and tumor-associated EVs (TAEs) are required.

**Methods:**

This study used quantifiable antibody-DNA conjugate-assisted quantitative methods combined with proximity ligation technology to detect TAEs. The antibody-DNA conjugate contained one antibody associated with three oligonucleotides for signal amplification. The antibody in the conjugate can recognize the surface tumor antigens of TAEs. Simultaneously, DNA in the conjugate is attached to the surfaces of TAEs and holds the signal amplification post, converting protein identities to DNA amplification for protein detection, even at the molecular level.

**Results:**

These findings revealed that TAEs can be quantitatively detected using DNA-mediated quantitative polymerase chain reaction (qPCR). Antibody-DNA conjugates were used to recognize the epithelial cell adhesion molecule (EpCAM) antigen on the TAE surface and quantify the antigen using qPCR for cancer analysis.

**Discussion:**

This method proposed a new quantitative detection approach for TAEs, which aim to identify specific EV-associated markers for diagnostic or therapeutic, this method could inspire a new idea for tumor diagnosis and detection of other diseases.

## 1 Introduction

Extracellular vesicles (EVs) are membrane-coated nanometer-sized vesicles released by cells into the bloodstream, cerebrospinal fluid, urine cell culture, and other bodily fluids (Thery et al., 2002). EVs move biological materials and messages from the original cell to other cells, affecting physiological and pathological processes such as metastatic niches and immunosuppression (Peinado et al., 2012; Hoshino et al., 2015; Liu et al., 2016; Costa-Silva et al., 2015; Chen G. et al., 2018; Poggio et al., 2019). Studies have proved that EVs could promote tumorigenesis by regulating immunity, promoting angiogenesis and metastasis in the tumor microenvironment (Liang et al., 2021).

EVs are highly heterogeneous among the surface proteins and contents that characterize the tissues of origin (Castillo et al., 2018; Larssen et al., 2017). Accordingly, EVs shed by both tumor cells into the peripheral circulation or other body fluids are promising biomarkers for cancer diagnosis due to their long circulation and enormous amount (Bhat et al., 2024). Recent reports have revealed that EVs are a promising biomarker for early disease detection (Castillo et al., 2018; Melo et al., 2015; Lee et al., 2017; Tavoosidana et al., 2011; Kang et al., 2012). In addition, EV-based biomarkers can directly influence clinical decision-making, provide doctors with more accurate diagnostic information, help formulate personalized treatment plans, and improve patient outcomes by improving the early detection rate of cancer and other diseases, strengthening the monitoring of effects during treatment, and predicting the disease progression and prognosis of patients.

Although the excellent clinical value, their use in personalized healthcare practice is not yet feasible due to their highly heterogeneous nature (Chen et al., 2023). Considering that tumor associated EVs (TAEs) are drowned in total EVs, methods to distinguish between normal EVs and TAEs are required for their application in disease detection. Currently, it is more challenging to detect EVs with sizes of less than a few hundred nanometers. Moreover, the isolation and characterization of EVs at a single-particle level is difficult. Nanosight-led nanoparticle tracking analysis has been applied in EV analysis, especially for EV quantification. However, it can be difficult to distinguish between normal EVs and TAEs, and even from other particles (Rupert et al., 2017; Shang and Gao, 2014).

Recently, antibodies and aptamers have gained popularity as new types of immuno-affinity moiety in cell labeling, cell surface modification, and cell-cell interaction (Zhu et al., 2013; Tan et al., 2013). Given the similarity between the cell membrane and EV membrane surface, TAEs in plasma are identified with aptamer-based methods with high affinity and specificity. Tumor-specific modifications, such as glycoxidation-induced neo-epitopes on proteins, have been shown to elicit specific immune responses, which could also serve as biomarkers in cancer detection (Chen J. et al., 2018; Zhang et al., 2019; Wan et al., 2017; Mir and Moinuddin, 2016). A modified proximity ligation assay (PLA) was developed to detect prostasomes as biomarkers for prostate cancer using four antibodies with attached DNA strands (Tavoosidana et al., 2011). Surface proteins of individual exosomes were analyzed using an antibody-based immune sequencing method and a proximity-dependent barcoding assay (Wu et al., 2019; Ko et al., 2021). Site-specific antibody-DNA conjugates were used in immuno-polymerase chain reaction (PCR) assays to detect Her2^+^ cells with greater sensitivity, and they can detect extremely rare Her2^+^ cells in a complex cellular environment (Kazane et al., 2012). A PLA for detecting proteins leveraged the amplification power of PCR by linking the presence of the target analytes to the production of a PCR amplicon that could be detected with extreme sensitivity (Robinson et al., 2016). By illustrating the biodistribution of melanoma-derived exosomes in mice (Peinado et al., 2012) and mammary (Hoshino et al., 2015; Fong et al., 2015) yuan, colorectal (A.R. and R.J.S., unpublished observations), pancreatic (Costa-Silva et al., 2015) and prostate (Smyth et al., 2015) cancer cells, show common metastasis sites of the same cancer type in humans (Xu et al., 2018), suggesting that EVs are equally applicable as biomarkers in other cancers.

Although various quantitative immuno-PCR-based techniques have been developed and used to detect various molecules (Chavan et al., 2020; Simonova et al., 2018; Sharma et al., 2019), in this study, one antibody and three oligonucleotides were joined together by streptavidin, a tetrameric protein that can bind biotinylated antibodies and biotinylated DNAs together. These quantifiable oligonucleotides in antibody-DNA conjugates can be used to quantify antibodies and antigenic proteins in TAEs. We provided a proof-of-principle that quantifiable antibody-DNA conjugate-assisted quantitative PCR (QDAC-qPCR) combined with proximity ligation technology can successfully detect TAEs. In this method, antibody-DNA conjugates were applied to detect TAEs, which combine affinity antibodies with amplifiable oligonucleotides. The monoclonal antibody in the conjugate assumes specificity to recognize the surface antigens of the TAEs, allowing accurate differentiation between normal cells and exosomes of tumor origin. In contrast, the DNA in the conjugate was attached to the surface of the TAEs. It holds the position of signal amplification, thereby converting protein quantity to DNA quantification for protein detection. Simultaneously, proximity ligation technology enables high-throughput quantification of exosomes in different tumor cells, making the method highly affinitive, sensitive, rapid hybridization, and accurate quantification.

## 2 Materials and methods

### 2.1 Materials

HCT116 cells (TCH-C185) were sourced from Hangzhou Haixing Biotechnology Company, and A549 and MD-231 cell lines were obtained from Z.W.Z.’s laboratory at Hangzhou Normal University. The primers and biotinylated oligo DNAs were synthesized by the Beijing Qingke Company. The biotinylated epithelial cell adhesion molecule (EpCAM) monoclonal antibody (ab79079) was procured from Abcam. Penicillin, Roswell Park Memorial Institute (RPMI)-1640 culture medium, 0.25% pancreatin (1×), and phosphate-buffered saline (PBS, 10×) were acquired from HyClone. Fetal bovine serum (FBS) was obtained from EVERY GREEN, and EVs were removed by ultracentrifugation. Streptavidin (100 μg/mL), radioimmunoprecipitation assay buffer, Bradford protein assay kit, and enzyme-linked immunosorbent assay (ELISA) kit were obtained from Beijing Solarbio Company. TB Green Premix Ex Taq (RR420A) for qPCR was obtained from TaKaRa Bio. The MagCapture^TM^ exosome isolation kit PS (299-77603) was sourced from Wako.

### 2.2 Exosome extraction method

When the cell density in the culture dish reached 85%, and the condition was good, the complete medium was discarded, the cells were gently rinsed with PBS three times, 7 mL of basal medium (+1% P/S) was added, and incubated in a cell culture incubator for 24 h. The supernatant of the cells was collected and centrifuged at 3,000 × *g* for 10 min to discard the cells and cellular debris. After transferring the cell supernatant to a new centrifuge tube, 1/4 of the cell supernatant was added to the exosome extraction reagent, mixed well, and incubated at 4°C overnight. The next day, the cells were centrifuged at 10,000 × *g* and 4°C for 1 h. The extent of precipitation was marked using a pen, and the supernatant was carefully discarded and aspirated as cleanly as possible. The precipitate was resuspended in 200 µL of PBS. The exosome suspension was centrifuged at 12,000 × *g* and 4°C for 2 min, and the supernatant was retained. The supernatant was transferred to an exosome purification filter column chamber and centrifuged at 3,000 × *g* for 10 min at 4°C. The liquid at the bottom of the tube contained purified exosomes. The obtained exosomes were divided into 100 µL per tube and stored at −80°C for a long time.

### 2.3 Exosome ultracentrifugation extraction

A549 and MD-231 cell lines were cultured for 24 h at 37°C in RPMI-1640 medium supplemented with 10% FBS (without EVs). The supernatant of the cell lines (50 mL) was harvested and centrifuged at 300 × *g* and 4°C for 5 min for the initial purification. The supernatant was centrifuged at 1,200 × *g* and 4°C for 20 min. Then, the supernatant was centrifuged at 10,000 × *g* and 4°C for 30 min to remove cell debris and microvesicles. Finally, EVs were isolated by ultracentrifugation at 100,000 × *g* and 4°C for 70 min in a type 70 Tirotor and suspended in 5 mL for subsequent labeling and detection.

### 2.4 Preparation of proximity antibody-DNA

Briefly, 2 μL bio-EpCAM (2.5 μM) and 2 μL bio-proximity C1 (2.5 μM) were mixed, 1 μL SA (2.5 μM) was added to the above mixture, incubated it for 30 min at room temperature, and 1 μL of cls-bio (2.5 μM) was sealed it for 20 min to obtain the antibody-DNA 1. Antibody-DNA 1 was diluted to 50 nM and stored in reserve. Antibody-DNA 1 was diluted to 50 nM and stored in reserve. Briefly, 2 μL of bio-proximity C1 (2.5 μM) was taken, following the same procedure as above, to obtain antibody-DNA 2. Afterward, 1 μL of each antibody-DNA 1 and 2 was incubated with 48 μL of A549 and HCT116 exosomes at 4°C overnight, respectively.

### 2.5 Preparation of SA-proximity ligation qPCR system

Briefly, 1 μL of bio-proximity C1 (10 μM), bio-proximity C2 (10 μM), 0.25 μL of SA (10 μg/mL) were placed in a centrifuge tube and incubated for 30 min at room temperature (at this time, the DNA concentration was 4.4 μM). The SA mixture was diluted to 1 nM and 100 pM and divided into four groups: bio-100 pM, bio-10 pM, 100 pM, and 10 pM. The SA mixture, proximity cnct, T4 DNA Ligase, and T4 DNA buffer (10×) were placed in a centrifuge tube. Sterilized water was added to bring the system volume to 20 μL, vortexed, mixed, and incubated at room temperature for 10 min. The spiking process was performed on ice.

All statistical analyses were performed using GraphPad Prism 9.0. All quantitative results are presented as the mean ± standard deviation (SD) of at least three independent replicates. Statistical significance between groups was assessed using a one-way analysis of variance (ANOVA).

## 3 Results

### 3.1 Synthesis of antibody-DNA conjugates

The 69-nucleotide biotinylated DNA and probes in this study (Table 1) were synthesized by the Beijing Qingke Company. Streptavidin is a tetrameric protein that binds four biotin molecules with an extremely high affinity. Streptavidin was employed to conjugate biotinylated anti-EpCAM antibody and biotinylated DNA, which was expected to bind one biotinylated EpCAM antibody and three biotinylated DNA strands. Initially, 10 µL of EpCAM antibody (10 µg/mL, approximately 0.06 µM) and 2 µL of biotinylated DNA (10 µM) were mixed. Then, 3 µL of streptavidin (100 µg/mL, approximately 1.6 µM) was added to the mixture and incubated for 30 min to form antibody-DNA conjugates. In this case, the molar ratio of antibody to streptavidin was approximately 1:80.

**TABLE 1:** DNA sequences were used in this study.

Oligo	Sequence (5′ to 3′)	Modification
EXOpcr-69-bio	CCAGGGAGTGATGGTTG GAATGAACCCGCTTCAG CAAGACTCACT*CTGAA GTATCCGATAGAACACGGC	5′Biotin
EXO-F	CCAGGGAGTGATGGTTGGAATG	—
EXO-R	GCCGTGTTGGCTCGGATAC	—
Probe-Rox	AGTGAGTCTTGCTGAAGCGG	5′Rox
Proximity C1	CGCATCGCCCTTGGACTACGA CTGACGAACCGCTTTGCCTGA CTGATCGCTAAATCGTG	—
Proximity C2	TCGTGTCTAAAGTCCGTTACC TTGATTCCCCTAACCCTCTTG AAAAATTCGG	5′PO4
Bio-proximity C2	TCGTGTCTAAAGTCCGTTACCT TGATTCCCCTAACCCTCTTG AAAAATTCGG	3′Biotin
Bio-proximity C1	GAACCGCTTTGCCTGACTGATC GCTAAATCGTG	5′Biotin
Proximity cnct	TACTTAGACACGACACGATTT AGTTT	—
Proximity F	CATCGCCCTTGGACTACGA	—
Proximity R	GGGAATCAAGGTAACGGACTTTAG	—
TaqMan SLC	TGACGAACCGCTTTGCCTGA	5′FAM 3′MGB
C1C2	CGCATCGCCCTTGGACTACGACTG ACGAACCGCTTTGCCTGACTG ATCGCTAAATCGTGTCGTGT CTAAAGTCCGTTACCTTG ATTCCCCTAACCCTC TTGAAAAATTCGG	—

*Hybridized with Probe-Rox, EXO-F, and EXO-R, are primers for bio-69; Proximities F and Proximities R are primers for bio- Proximity C1, bio- Proximity C2, and Proximity cnct after ligation, and for C1C2.

**FIGURE 1:**
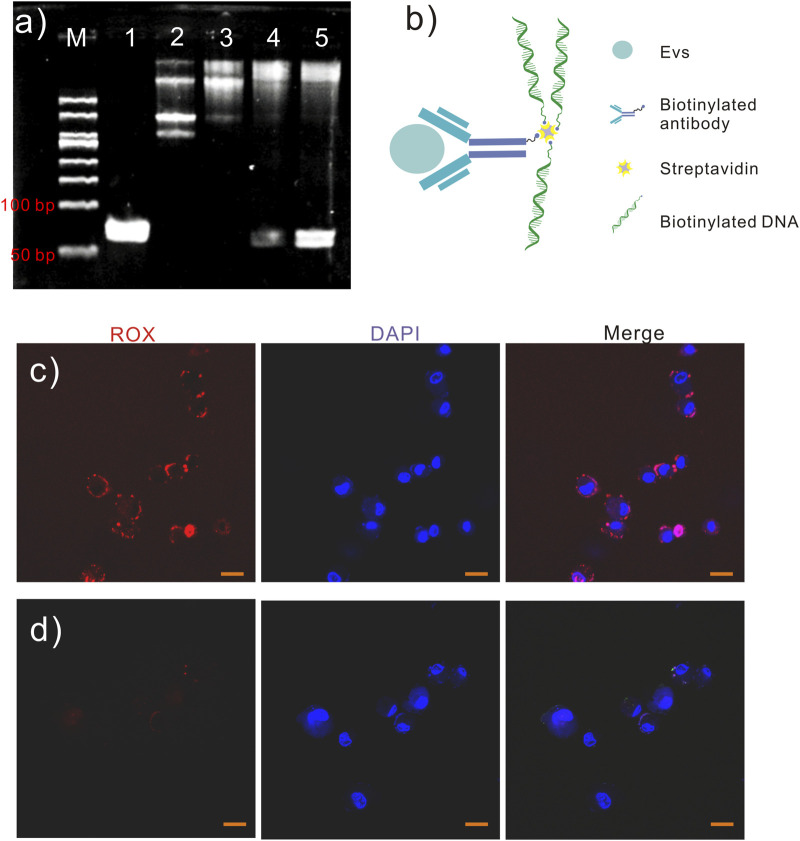
Antibody-DNA conjugates. **(A)** Different numbers of biotinylated DNAs (10 μM) were bonded to streptavidin (1.6 μM), and the reactions were analyzed using polyacrylamide gel electrophoresis (8%, w/w). Lane 1, DNA 1 μL; lane 2, DNA 2 μL and streptavidin 1 μL; lane 3, DNA 3 μL and streptavidin 1 μL; lane 4, DNA 4 μL and streptavidin 1 μL; lane 5, DNA 5 μL and streptavidin 1 μL. **(B)** Schematic diagram of antibody−DNA conjugates. **(C)** A549 cells were labeled with antibody–DNA conjugates and probed for red fluorescence. However, A549 cells labeled with DNA are evident **(D)** without red fluorescence. Scale bar in **(C, D)**, 20 µm.

Antibody-DNA conjugates were established and applied to detect TAEs. The monoclonal antibody in the conjugate assumes specificity to recognize the surface antigens of TAEs. Simultaneously, DNAs in the conjugate are attached to the TAE surfaces and serve as signal amplification for PCR. The antibody and DNAs in the conjugate were joined using streptavidin, a tetrameric protein that can bind biotinylated antibodies and biotinylated DNAs. When the number of biotinylated DNAs was less than the number of streptavidin subunits, four DNA ladders appeared in the gel electrophoresis, indicating that streptavidin molecules bound one to four biotinylated DNAs (Figure 1A). When the number of biotinylated DNA exceeds that of biotinylated antibodies, it is feasible that the biotinylated antibodies occupy one streptavidin subunit with three biotinylated DNAs. When the number of biotinylated DNA molecules exceeded that of biotinylated antibodies, one biotinylated antibody was conjugated with three biotinylated DNAs in one streptavidin molecule.

Because the binding behavior between streptavidin and biotinylated molecules is random, the ratio of biotinylated antibody binding to streptavidin molecules can be regarded as a Poisson distribution.

According to the Poisson distribution (Equation 1),

$$P\left(n,\lambda \right)=\left(\lambda^{n}e^{\left(-\lambda \right)}\right)/n!$$ (1)

The probability of more than two biotinylated antibodies binding to the streptavidin molecule was P (n > 1) (Equations 2–5), where n is the number of biotinylated antibodies bound to the streptavidin molecule.

$$P\left(n>1,\lambda \right)=1-P\left(n=0,\lambda \right)-P\left(n=1,\lambda \right)$$ (2)

$$P\left(n=0,\lambda \right)=e^{\left(-\lambda \right)}$$ (3)

$$P\left(n=1,\lambda \right)=\left(\lambda ∙e^{\left(-\lambda \right)}\right)$$ (4)

$$P\left(n>1,\lambda \right)=1-e^{\left(-\lambda \right)}-\left(\lambda ∙e^{\left(-\lambda \right)}\right)$$ (5)

when λ (the ratio of biotinylated antibodies to streptavidin molecule) is 1:80, therefore

$$P\left(n>1,\lambda \right)=7.7\times 10^{-5}$$ (6)

In this study, 10 µL of EpCAM antibody (10 µg/mL, approximately 0.06 µM) and 3 µL of streptavidin (100 µg/mL, approximately 1.6 µM) were incubated, resulting the molar ratio (λ) of biotinylated antibody antibodies to streptavidin molecules of 1:80. According to this calculation (Equation 6), the probability that more than two biotinylated antibodies bind to the streptavidin molecule is 7.7 × 10^−5^. Most biotinylated antibodies occupied only one subunit of streptavidin, while three biotinylated DNAs occupied the other three streptavidin subunits. The antibody-DNA conjugate contained one antibody and three DNA molecules. The antibody-DNA conjugate realized the preliminary amplification of the antibody signal through three DNA molecules. Additionally, the antibody signal was magnified by DNA amplification. Moreover, the proteins can be quantified by quantifying the DNA using qPCR.

The EpCAM is a 40 KD transmembrane glycoprotein that functions as an adhesion molecule (Robinson et al., 2016) and plays an important role in regulating cell adhesion and signaling pathways in cancer (Peinado et al., 2012). EpCAM-specific monoclonal antibodies (mAbs) have been used in treating human colorectal cancer since 1900, with a 30% increase in five-year survival and a 27% reduction in recurrence rates within 7 years of treatment (Hoshino et al., 2015). Moreover, EpCAM-specific antibodies were first approved for treating colorectal cancer in 1995 (Fong et al., 2015). Normally, EpCAM is expressed at low levels in epithelial tissues; however, it is highly expressed in most precancerous tissues and almost all adenocarcinomas, including colorectal, gastric, breast, and pancreatic cancer (Costa-Silva et al., 2015). Since EpCAM is a common biomarker for cancer, an EpCAM monoclonal antibody was used to recognize TAE surface antigens. In this study, antibody-DNA conjugates (Figure 1B) were used to identify the specific antigen in TAEs using the antibody and amplify the signal using DNA with PCR. Antibody-DNA conjugates were used to label specific antigens in TAEs or cells. Once antibody-DNA conjugates were established, as described above, they were used to label specific cells. Biotinylated anti-EpCAM antibody (10 µg/mL) 10 and 2 µL of biotinylated DNA (10 µM) were added to 3 µL of streptavidin solution (100 µg/mL) for 30 min. The A549 cells were labeled with EpCAM antibody-DNA conjugates for 2 h at 37°C to recognize the EpCAM protein on the cell surface after blocking cells with bovine serum albumin and salmon sperm. Labeled A549 cells carried the EpCAM antibody-DNA conjugates and were soaked in a solution containing Probe-Rox (Red, Table 1) that hybridized to the DNA of conjugates. Then, A549 cells were rinsed five times to remove the superfluous conjugates and Probe-Rox. The fluorescence of individual cells was detected using confocal microscopy to determine the existence of antibody-DNA conjugates on the cell members (Figure 1C). However, no obvious fluorescence was observed in the cells labeled with DNA alone (Figure 1D). This result revealed that antibody-DNA conjugates could specifically bind to antigens on the tumor cell membrane surface. Consequently, we can reasonably speculate that these antibody-DNA conjugates can specifically bind to the antigen on the surface of the EV membrane for TAE detection and quantification.

### 3.2 EVs labeling and detection of EpCAM^+^ cancer cell

EpCAM is a tumor cell exosome marker that differentiates between normal and tumor exosomes. In this study, three EpCAM^+^ cancer cell lines, HCT116, A549, and MD-231, were selected as models for different expression levels of EpCAM to explore the feasibility, accuracy, and sensitivity of this exosome detection method.

Subsequently, we performed EV labeling and detection. This study extracted exosomes from A549 and MD-231 cell lines by ultracentrifugation for subsequent labeling and detection. Antibody-DNA conjugates were applied to recognize the antigen on the TAE surface and to detect the antigen using PCR to diagnose cancer.

**FIGURE 2:**
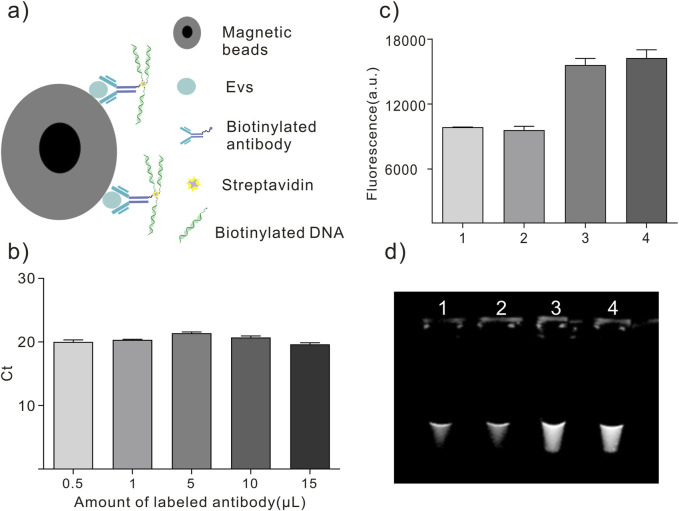
EVs labeling and detection. **(A)** Schematic diagram of antibody-DNA conjugates labeled on EVs. **(B)** Different volumes of antibody-DNA conjugates were mixed with EVs to ensure efficient labeling. **(C, D)** Antibody-DNA conjugate-labeled EVs were detected using PCR. 1. PBS; 2. EVs in PBS; 3. Antibody-DNA conjugate-labeled EVs in PBS; 4. DNA in PBS.

The EVs in 500 mL supernatant of the A549 cell line were isolated by ultracentrifugation for 70 min at 100,000 × *g* and 4°C and suspended in 5 mL PBS to confirm the feasibility of the labeling and detection method. This study used the MagCapture exosome isolation kit phosphoesteryl serine (PS) to capture EVs for labeling (Figure 2A). Multiple groups of 6 μL magnetic beads with exosome capture were separately added to 100 μL of EV solution according to the protocol. The EVs in the solution were captured and fixed on the surface of the magnetic beads. The advantage of this kit is that the captured EVs can be eluted after labeling with an elution buffer.

Then, different volumes of antibody-DNA conjugates were mixed with EVs on the 6 µL magnetic beads surface for 2 h. After collecting the beads and washing them five times with washing buffer, the antibody-DNA conjugate-labeled EVs were released into the solution via the elution buffer and detected using qPCR. Figure 2B displays that the cycle threshold (Ct) value did not significantly change in the five EV groups. An antibody (10 μL, 10 mg/mL) was used to ensure labeling efficiency for subsequent EV labeling. PCR detected the antibody-labeled EVs to ensure the feasibility of this method. Figures 2C, D depict that antibody-DNA conjugate-labeled EVs could be detected using PCR.

### 3.3 EVs labeled with the antibody-DNA conjugate and detected using qPCR and ELISA

The total EVs in the supernatant were captured by Tim4 binding PS using the MagCapture exosome isolation kit PS. A DNA-conjugated EpCAM antibody was added to label the EVs and incubated for 120 min at 4°C. Then, the magnetic beads were collected and washed five times with a washing buffer, and the supernatant was discarded. Finally, the antibody-labeled EVs were released into the solution via the exosome elution buffer in the kit. Labeled EVs were quantitatively detected using qPCR to amplify the DNA labeled on the surface of EVs.

Next, we constructed antibody-DNA conjugates using previously described methods for labeling and detecting EVs on the surface of magnetic beads. The EpCAM antibody-DNA conjugates were used to detect EpCAM^+^ EVs quantitatively. The total protein concentration of the EVs from the beads was 120 µg/mL, as determined using the Bradford protein assay kit. EpCAM^+^ EVs were labeled with EpCAM antibody-DNA conjugates and eluted from the beads. DAC-qPCR and ELISA quantitatively detected gradient dilutions of labeled EVs.

The PCR solution was mixed with 2 µL of labeled EVs, and DNAs anchored on the EVs surface were detected using qPCR. The qPCR results demonstrated that when the concentration of total exosomal protein was 1.20 × 10^−3^ μg/mL, the CT value reached the maximum detection limit.

**FIGURE 3:**
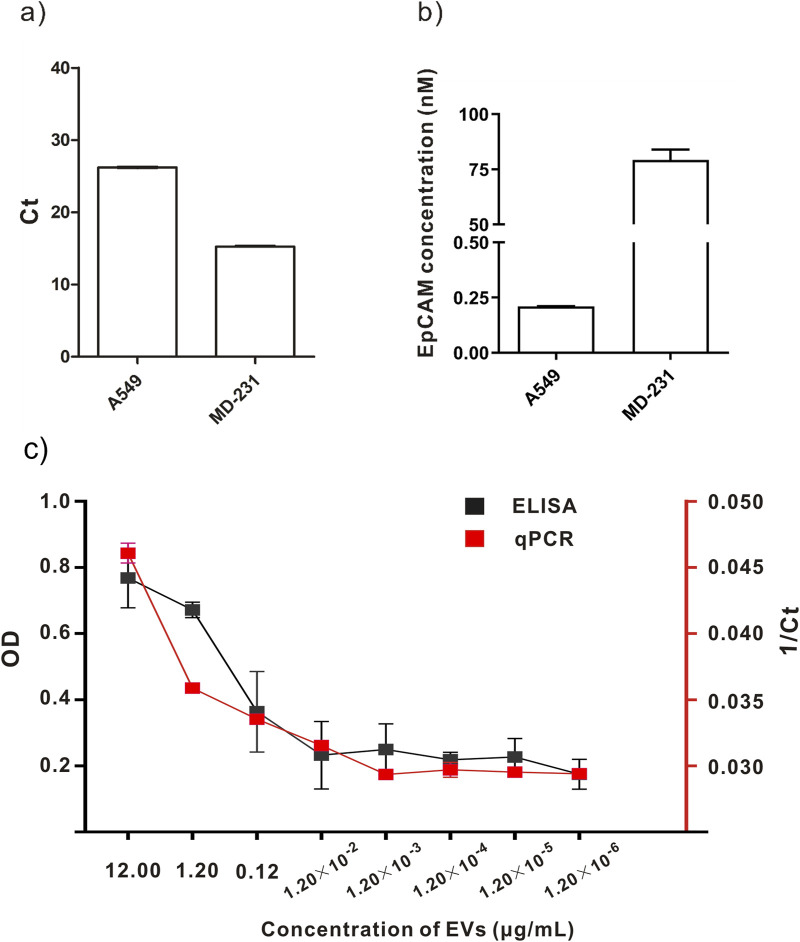
Comparison of labeling and detection of EVs from different cell lines using different methods. **(A, B)** Quantitative detection of A549 and MD-231cell lines. **(C)** Comparison between qPCR and ELISA results.

Concurrently, a series of dilutions of 100 µL of labeled EVs were added to the ELISA plate. Horseradish peroxidase (HRP)-labeled secondary antibodies were used to detect the antibodies on EVs using the optical density (OD). The ELISA results revealed that the concentration of total exosomal protein was 1.20 × 10^−2^ μg/mL, and the OD value reached the maximum detection limit (Figure 3C).

We concluded that the DAC-qPCR method is superior to ELISA in detecting the concentration of EVs compared to the experimental results of EV concentration measured by DAC-qPCR and ELISA. Additionally, fewer samples were used in the DAC-qPCR method than in the ELISA method. In the specific experimental process, the DAC-qPCR method only requires several microliters of sample, while ELISA samples require hundreds of microliters. The DAC-qPCR combines affinity antibodies with conjugated amplifiable oligonucleotides, converting protein identities to DNA amplification for protein detection even at low levels of molecules or molecular complexes.

Besides, we also quantitatively detected EpCAM^+^ EVs by DAC-qPCR using EXOpcr-69-biot DNA as reference material. The DNA concentration of antibody-DNA conjugates anchored on the EVs surface was 2.41 ± 0.48 nM by DAC-qPCR. Furthermore, the DNA to the monoclonal antibody ratio was 1:3, and this could be converted that the antibody concentration or antigen concentration was 0.80 ± 0.16 nM, presenting that EpCAM protein in total EVs (120 µg/mL) was 0.80 ± 0.16 nM or 23.4 ± 4.6 ng/mL (molecular weight of EpCAM is 29.1 kD).

### 3.4 Quantitative detection of different cell lines

This quantitative method detected the concentration of EVs in the supernatants of A549 cells and MD231 cells. Figures 3A, B depict that CT values of MD-231 cells were lower, indicating that this method is applicable to detect EVs from different tumors. The DNA concentration of antibody-DNA conjugates anchored on A549 cells derived EVs surface was 0.21 ± 0.005 nM using DAC-qPCR. The DNA concentration of antibody-DNA conjugates anchored on MD-231 derived cells EVs surface was 79.23 ± 8.1 nM using DAC-qPCR. Moreover, the EpCAM monoclonal antibody was used to recognize EpCAM in TAEs using this method; accordingly, the number of DNAs reflected the number of EpCAM in TAEs. We conclude that DAC-qPCR could be used to quantify more cancer biomarkers in TAEs with different mAbs.

### 3.5 Detection of exosomes by proximity ligation qPCR with antibody-DNA conjugates

**FIGURE 4:**
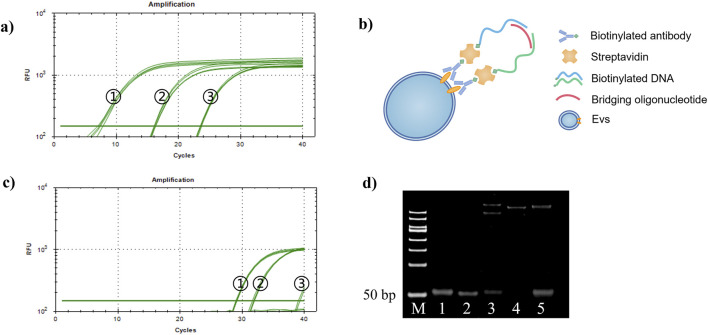
Proximity ligation concentration exploration. **(A)** Proximity ligation concentration exploration diagram. (1) 10 nM, (2) 1 nM, and (3) 100 pM. **(B)** Schematic diagram of proximity ligation of antibody-DNA conjugate-labeled exosomes. **(C)** SA-DNA1 and SA-DNA2 proximity ligation qPCR plots. (1) bio-100 pM, (2) bio-10 pM, (3) 100 pM, and (4) 10 pM; 10 pM was undetected due to the low concentration. **(D)** SA versus DNA ligation agarose gel electrophoresis plots (2%, w/w). Lane 1: bio-proximity C1; lane 2: bio-proximity C2; lane 3: SA + bio-proximity C1; lane 4: SA + bio-proximity C2; lane 5: SA + bio-proximity C1 + bio-proximity C2.

In this study, we investigated the recognition and detection abilities of antibody-DNA conjugates on tumor exosomes using the proximity ligation technique. We used a biotin anti-EpCAM antibody and two different DNA templates, C1 and C2, to construct two kinds of antibody-DNA conjugates, allowing the two antibody-DNA conjugates to label the tumor exosomes. Simultaneously, we synthesized a section of oligonucleotides with half of the complementary sequences with these two DNAs as bridging oligonucleotides to bring the distance between the two and achieve proximity ligation (Figure 4B). Initially, the minimum concentration required for qPCR was determined. We diluted DNA templates C1 and C2 to 1 nM, 10 nM, 1 nM, 100 pM, and 10 pM, respectively. Only the concentrations of 10 nM, 1 nM, and 100 pM were within the instrument’s detection range, while the other two concentrations were out of the range and did not result in an amplification curve (Figure 4A). The qPCR results indicated that the lowest starting concentration of DNA that the instrument could detect to achieve proximity ligation was 100 pM, suggesting that we could choose 100 pM or lower for subsequent experiments. Using a 100 pM concentration as the reference concentration for streptavidin (SA)-proximity ligation exploration, bio-proximities C1 and C2 were conjugated to SA, and the biotin sites were closed with biocytin to obtain two conjugates, SA-C1 and SA-C2. Electrophoresis results (Figure 4D) demonstrated that lanes 3, 4, and 5 had bands at >500bp, indicating that SA was conjugated to bio-proximities C1 and C2 and biocytidine, respectively. Bio-proximities C1 and C2 indicate that SA was successfully ligated with bio-proximities C1 and C2, respectively, and SA could be ligated with both simultaneously. This indicated that the SA-DNA conjugate was successfully prepared. We mixed SA-C1 and SA-C2, added bridging oligonucleotides to prepare the proximity ligation reaction system, and configured the qPCR reaction system for online detection. The Ct value of the bio-100 pM group was approximately 26.96, the Ct value of the bio-10 pM group was approximately 29.65, and the other two control groups had no Ct value. This study concluded that SA could reduce the distance between two DNAs to reduce the detection limit of qPCR when the DNA concentrations were 100 and 10 pM (Figure 4C).

**FIGURE 5:**
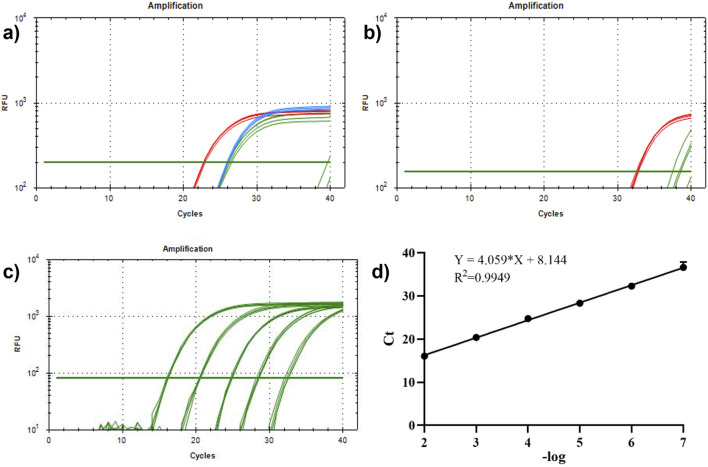
Detection of antibody-DNA conjugate proximity ligation of tumor exosomes and plotting the proximity ligation standard curve. **(A)** Detection of antibody-DNA conjugate proximity ligation of HCT116 exosomes. Red line: 100 pM experimental group; blue line: 10 pM experimental group; green line: 100 and 10 pM control groups; **(B)** antibody-DNA conjugate proximity ligation detection of A549 exosomes. Red line: 100 pM experimental group; green line: 100 pM control group. **(C)** qPCR curve of C1C2. **(D)** Standard curve of C1C2. n = 3, x̅ ± SD.

Next, antibody-DNA conjugates 1 and 2 were constructed with two DNAs, bio-proximity C1 and C2, respectively, to label the tumor exosomes. Figures 5A, B display that the Ct values of the experimental group were smaller than those of the control group at 100 and 10 pM concentrations. This indicated that the antibody-DNA conjugates 1 and 2 successfully labeled the exosomes of HCT116 and A549 cells and could be detected by proximity ligation at a concentration of 10 pM, implying that the method is capable of significantly increasing sensitivity and lowering the limit of detection. We designed and synthesized a DNA sequence C1C2 containing C1 and C2 to quantify the extracted tumor exosomes. Template C1C2 was diluted 10-fold, and the qPCR system was configured and tested on a machine (Figure 5C). The obtained data were analyzed, and the C1C2 standard curve was plotted using the negative logarithm of the dilution on the *X*-axis and the Ct value on the *Y*-axis (Figure 5D). According to the C1C2 standard curve equation Y = 4.059X + 8.144 and Poisson distribution, the concentrations of HCT116 and A549 exosomes extracted by ultrafiltration were 8.57 × 10^−5^/mL and 6.96 × 10^−7^/mL, respectively. When the concentration of DNA was 100 pM, HCT116 and A549 exosomes were 2.53 × 10^−4^ and 1.10 × 10^−6^ exosomes/mL, respectively. A comparison between the two extraction methods revealed that the kit extraction method had an order of magnitude improvement in the extraction efficiency of the same exosome compared to the ultrafiltration method. Overall, our constructed antibody-DNA conjugates could label tumor exosomes and reduce the detection limit of qPCR using the proximity ligation method.

## 4 Conclusion and discussion

At present, circulating tumor cells (CTCs), circulating tumor DNA (ctDNA), and exosomes have become the three major branches of liquid biopsy (Ye et al., 2019). Compared to CTCs and ctDNA, exosomes have an advantage in liquid biopsy. First, the presence of a large number of exosomes (∼10^9^ per mL) in biofluids makes it relatively easy to obtain vesicles, whereas only a few to dozens of CTCs are present in a 1 mL blood sample, making their capture very difficult (Alix-Panabières and Pantel, 2021). Secondly, exosomes are secreted by living cells, and their parent cells are rich in biological information. ctDNA, on the other hand, is a fragment of DNA produced by apoptosis or death, which reflects a limited amount of information about apoptotic or dead tumor cells (Hoshino et al., 2015). Therefore, exosomes are more representative than ctDNA in tumor detection. Third, the lipid bilayer of exosomes makes them inherently stable and circulate under physiological conditions, even in harsh tumor microenvironments. The high biostability of exosomes allows for long-term storage for the isolation and detection of exosomes (Yu et al., 2021). However, due to their nanoscale size and intrinsic heterogeneity, exosomes face difficulties in their application in liquid biopsy (He et al., 2018). In addition, tumor-derived exosomes make up only a small fraction of all exosomes in body fluids, so sensitive and specific detection is a prerequisite for the development of exosome-based cancer diagnostics.

Using technologies for clinical diagnosis as soon as possible is inevitable to eliminate the interference of normal EVs, focusing on analyzing TAE subgroups. The antibody is an excellent targeting molecule that proactively identifies the antigen on the surface of TAEs; however, it has a drawback in signal amplification. In this study, we constructed antibody-DNA conjugates for targeting, signal amplification, and signal amplification. Subsequently, a method for quantitatively detecting TAEs was established using antibody-DNA conjugates and qPCR analysis. Proximity ligation methods were also used to reduce the quantitative detection limit. This study proposed a new quantitative detection method for TAEs based on tumor biomarkers, which can be applied to existing qPCR platforms. The application of this method provides a new idea for tumor diagnosis and the detection of other diseases. Nevertheless, There are many limitations of the study, such as the lack of functional validation of identified biomarkers, the small sample size and the absence of validation across independent patient cohorts or external datasets. Due to the characteristics of complex biofluid circumstances and small size of EVs, current isolation and characterization methods commonly used for single EVs are still facing many challenges, such as time consumption, low accuracy (Kang et al., 2012), these might affect the reproducibility of the findings. In addition,the findings are preliminary and require further validation.

## Data Availability

The original contributions presented in the study are included in the article/Supplementary Material, further inquiries can be directed to the corresponding authors.

## Supplementary material

SUPPLEMENTARY FIGURE S1 Characterization of HCT116 exosomes **(A)** Transmission electron micrograph (200 nm). **(B)** Particle size distribution graph. **(C)** BCA standard curve. **(D)** Protein immunoblotting graph.
