# Supplementary figures and images for: Labeling tumor-associated extracellular vesicles with antibody-DNA conjugates for quantitative analysis
Source: Front Mol Biosci. 2025 Jan 22;12:1531108. doi: 10.3389/fmolb.2025.1531108 (PMC11794122; doi:10.3389/fmolb.2025.1531108)

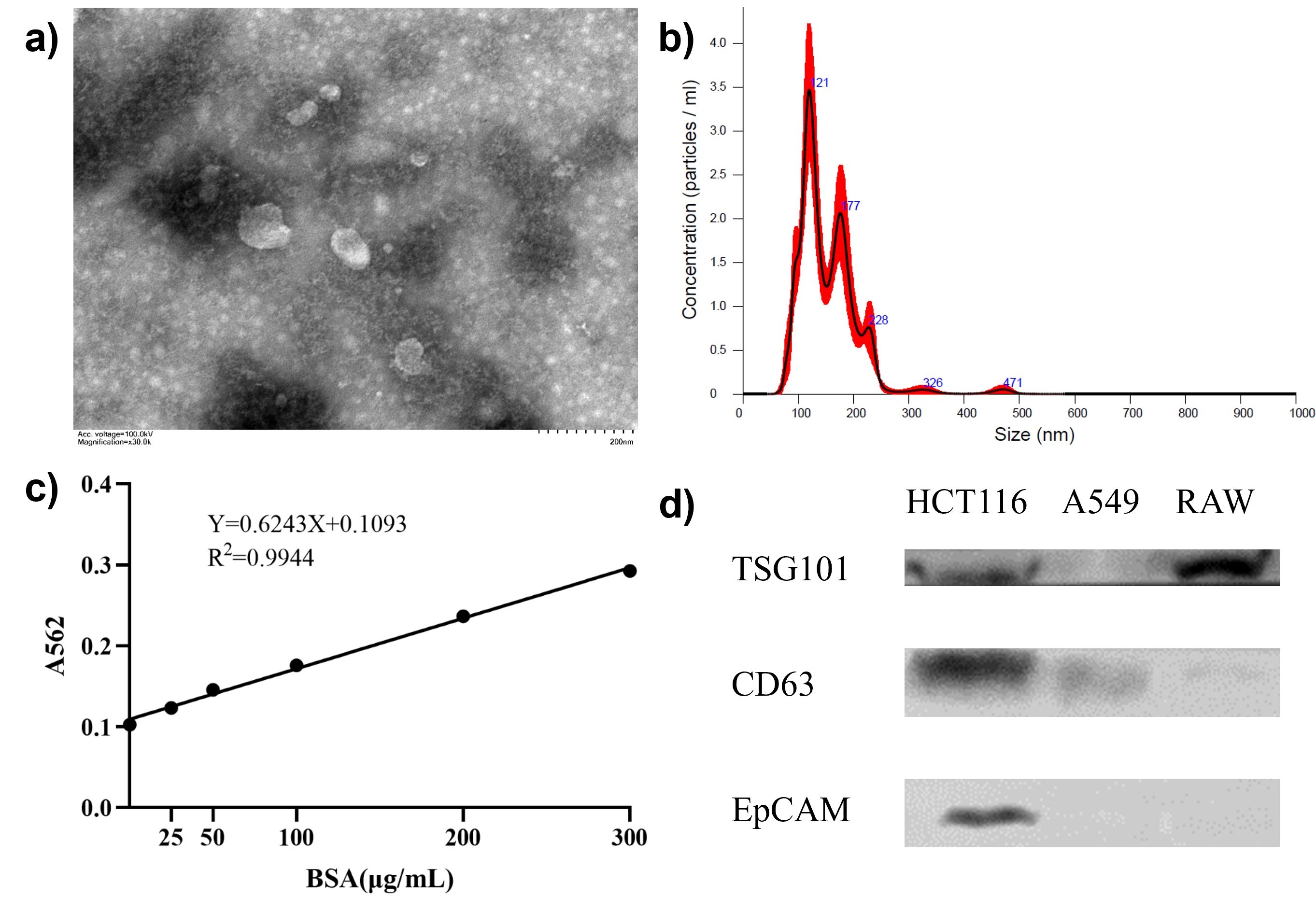

Supplement: Supplementary file 1 [file Image1.tif]
