# Supplementary material for: Labeling tumor-associated extracellular vesicles with antibody-DNA conjugates for quantitative analysis
Source: Front Mol Biosci. 2025 Jan 22;12:1531108. doi: 10.3389/fmolb.2025.1531108 (PMC11794122; doi:10.3389/fmolb.2025.1531108)
Supplement: Supplementary file 2 [file DataSheet1.docx]

**Figure legend**

Figure S1. Characterization of HCT116 exosomes (a) Transmission electron micrograph (200 nm). (b) Particle size distribution graph. (c) BCA standard curve. (d) Protein immunoblotting graph.

Supporting information

The cancer cell lines HCT116 and A549 and the macrophage cell line RAW 264.7 were cultured to collect extracted exosomes and characterized using transmission electron microscopy (TEM), nanoparticle tracing, and protein immunoblotting for exosome morphology, size, and surface protein expression. We characterized the obtained exosomes, which were elliptical and completely shaped in the TEM image (Figure S1a). The nanoparticle tracer analysis (NTA) results indicated that the particle size of exosomes was approximately 120 nm, and the concentration was approximately 2.57 × 109 particles/mL, which was within the normal range for exosomes (Figure S1b). We subjected the exosomes to protein cleavage and plotted the standard curve using the BCA（bicinchoninic acid）method (Figure S1c). We obtained the standard curve as Y = 0.6243X + 0.1093 and calculated the protein concentration using the standard curve to determine the amount of western blot (WB) samples. We selected CD63, TSG101, and EpCAM as proteins related to WB. The CD63 is a transmembrane protein on exosomes, TSG101 is a protein related to the ESCRT complex, which drives membrane formation and rupture, and EpCAM is a tumor cell exosome marker that differentiates between normal and tumor exosomes. Raw exosomes express TSG101 and CD63, HCT116 expresses all three proteins, and A549 only expresses CD63 (Figure S1d), possibly due to low protein concentration or insufficient protein expression. These results revealed that exosomes were successfully extracted.
